# Reactive Astrocytes in Glial Scar Attract Olfactory Ensheathing Cells Migration by Secreted TNF-α in Spinal Cord Lesion of Rat

**DOI:** 10.1371/journal.pone.0008141

**Published:** 2009-12-03

**Authors:** Zhida Su, Yimin Yuan, Jingjing Chen, Li Cao, Yanling Zhu, Liang Gao, Yang Qiu, Cheng He

**Affiliations:** Institute of Neuroscience and Key Laboratory of Molecular Neurobiology of Minister of Education, Neuroscience Research Center of Changzheng Hospital, Second Military Medical University, Shanghai, China; Emory University School of Medicine, United States of America

## Abstract

**Background:**

After spinal cord injury (SCI), the formation of glial scar contributes to the failure of injured adult axons to regenerate past the lesion. Increasing evidence indicates that olfactory ensheathing cells (OECs) implanted into spinal cord are found to migrate into the lesion site and induce axons regeneration beyond glial scar and resumption of functions. However, little is known about the mechanisms of OECs migrating from injection site to glial scar/lesion site.

**Methods and Findings:**

In the present study, we identified a link between OECs migration and reactive astrocytes in glial scar that was mediated by the tumor necrosis factor-α (TNF-α). Initially, the Boyden chamber migration assay showed that both glial scar tissue and reactive astrocyte-conditioned medium promoted OECs migration in vitro. Reactive astrocyte-derived TNF-α and its type 1 receptor TNFR1 expressed on OECs were identified to be responsible for the promoting effect on OECs migration. TNF-α-induced OECs migration was demonstrated depending on activation of the extracellular signal-regulated kinase (ERK) signaling cascades. Furthermore, TNF-α secreted by reactive astrocytes in glial scar was also showed to attract OECs migration in a spinal cord hemisection injury model of rat.

**Conclusions:**

These findings showed that TNF-α was released by reactive astrocytes in glial scar and attracted OECs migration by interacting with TNFR1 expressed on OECs via regulation of ERK signaling. This migration-attracting effect of reactive astrocytes on OECs may suggest a mechanism for guiding OECs migration into glial scar, which is crucial for OECs-mediated axons regrowth beyond the spinal cord lesion site.

## Introduction

Damage to adult mammalian central nervous system (CNS) leads to persistent functional deficits for the lack of axonal regeneration and reconnection with correct synaptic targets. The failure of spontaneous anatomical and functional repair is due not merely to the intrinsic incapacity of the neuron to regenerate but rather to the presence of a hostile environment in the lesion site. As the major cell type in CNS, astrocytes provide a variety of critical supportive functions that maintain neuronal homeostasis. When the CNS is damaged, astrocytes undergo an injury response and become reactive, characterized by hyperplasia, hypertrophy and an massive up-regulation of intermediate filament (IF) proteins, and leads eventually to the formation of a dense glial scar network at the lesion site [1]. The glial scar which composed primarily of reactive astrocytes has long been implicated as a major impediment to axon regeneration and functional outcome after SCI and other forms of CNS injury [2], [3]. It constitutes a mechanical obstacle and a biochemical barrier to preventing successful regeneration, as several classes of growth inhibitory molecules are upregulated and have been shown to contribute to the failure of axon regeneration [2], [4], [5], [6]. On the other hand, increasing evidence indicates that glial scar might also possess several important beneficial functions such as stabilizing fragile CNS tissue after injury [7], [8], [9]. After injury, reactive astrocytes form a dense scar tissue that has been suggested to seclude inflammatory cells, demarcate the lesion area, and separate the injured tissue from its surroundings [9]. Astrocytes have an important scavenging activity, which is crucial for regulating excessive levels of glutamate, K^+^ and other ions [10]. Moreover, the glial scar is reported to fill the gaps in the lesion area, creating a scaffold for the vascularization network [11].

Olfactory ensheathing cells (OECs) are the glial cells that derive from the olfactory placode and envelop olfactory axons in the course of migration from the olfactory epithelium to the bulb [12]. Owing to the axonal growth-promoting properties and the superior ability to interact with astrocytes, OECs transplantation has emerged as a promising experimental therapy to treat axonal injuries and been shown to induce anatomical and functional repair of lesions of spinal cord [13], [14], [15]. After SCI, the reestablishment of neural connections depends not only on the ability of nerve fibres to regeneration but also on the provision of a pathway along which they can elongate to reach appropriate destinations. Transplanted OECs have been shown to migrate with regenerating axons through an unfavorable CNS environment [16], [17], [18], [19] and to mingle well with astrocytes in adult brain [20], [21]. Interaction with astrocytes at the lesion site results in the formation of an OEC channel between the host astrocytic pathways on either side of the lesion, being devoid of inhibitory molecules and providing a pathway for the severed axons to regenerate successfully across the lesion and reach tissue targets [15]. However, the mechanism underlying OECs migration into the lesion region remains elusive.

Here, we provided the first evidence that reactive astrocytes attract OECs migration by secreted TNF-α not only *in vitro* but also in damaged spinal cord, suggesting a mechanism for guiding OECs migration into glial scar, which is crucial for OECs-mediated axons regrowth beyond the lesion site.

## Materials and Methods

### Ethical Statements

All animals in this study were handled in strict accordance with the recommended NIH guidelines for care and use of animals for scientific purposes and were approved by the Animal Experimentation Ethics Committee of Second Military Medical University.

### Primary cell cultures

Primary OECs were prepared from olfactory bulb of adult wild-type or GFP-transgenic Sprague-Dawley rats and purified by differential cell adhesiveness [22]. Briefly, OECs were extracted from olfactory nerve layer by trypsin treatment and plated on uncoated 25-cm^2^ culture flask two times, each for 36 h at 37°C in 5% CO2. The non-adhesive cell suspension was collected and then seeded onto disks pre-coated with poly-L-lysine (PLL, 0.1 mg/ml), and incubated with serum-containing DMEM/F-12 supplemented with 2 µM forskolin (Sigma) and 10 ng/mL bFGF (Sigma). OECs purity was assessed by staining with S100. The percentage of S100-positive cells in our culture system is more than 95%.

Highly enriched primary astrocyte cultures were prepared from the cerebral cortex of 2-day-old neonatal rat as described previously [23] with slight modifications. After removal of the meninges, the cerebral cortex was dissociated into a single-cell suspension by trypsinization and mechanical disruption. The cells were seeded on poly-L-lysine (PLL, 0.1 mg/ml, Sigma) coated culture flasks and incubated in DMEM/F-12 containing 10% foetal calf serum. The culture medium was replaced at 24 hours and every 3 days thereafter. After 8–10 days, the cultures became confluent and loosely attached microglia and oligodendrocyte precursor cells were removed from the cell monolayer by shaking the flasks on a rotary shaker at 260 rpm for 18–20 hours at 37°C. Astrocytes were subsequently detached using trypsin-EDTA and plated into PLL-coated 12-well plates or onto PLL-coated coverslips. The percentage of GFAP-positive cells in our culture system is more than 98%. Primary astrocyte cultures were stimulated chemically with lipopolysaccharide (LPS, 10 µg/mL) to induce astrocyte activation.

### Spinal cord injury model

Adult male Sprague-Dawley (170–200 g) rats were used for spinal cord injury study. Animals were maintained in a conventional animal facility that was kept at a 12∶12 h light–dark cycle with water and food provided *ad libitum*.

Surgical procedures were performed as previous protocol [24] with a slight modification. Animals were anaesthetized with 2% pentobarbital sodium. Laminectomy was performed to expose the dorsal surface of the T7-9 segment, followed by a spinal right hemisection at T8 using a fine corneal blade (cut twice in the same place to ensure complete section). Then, the dorsal back muscle and skin were sutured in layers. Postoperatively, animals were kept at 22–25°C on highly absorbent bedding, injected with cefazolin sodium (40 mg/day) for up to 1 week, and received manual bladder expression twice daily until reflexive bladder control returned.

### Preparation of culture conditioned medium

Astrocytes and reactive astrocyte-conditioned serum-free medium was obtained by growing confluent astrocytes and LPS-treated astrocytes for 48 hr in condition-defined medium that consisted of DMEM/F-12 supplemented with 1% N2 (vol/vol), 10.1 ng/ml T3, 400 ng/ml T4, 0.035% bovine serum albumin (BSA) and 20 µM leupeptin, respectively. The culture-conditioned serum-free medium prepared in this way was filtered (0.22 µm filter) and used for Boyden chamber migration assay.

### Boyden chamber migration assay

To measure the motility of a group of OECs, Boyden chamber migration assay was performed according to a previously described protocol [22]. In brief, the polyethylene terephthalate filter membranes were coated with laminin. OECs were detached by trypsin/EDTA and then seeded onto the upper chamber at a density of 4×10^5^ cells in 250 µl of culture medium containing 1% serum per well. The upper chambers were inserted into the tissue-culture wells and 750 uL culture medium containing 1% serum, defined medium (consisted of DMEM/F-12 supplement with 1% N2 (vol/vol), 10.1 ng/mL T3, 400 ng/mL T4, 0.035% bovine serum albumin (BSA) and 20 µM Leupeptin) or condition-defined medium were added to lower chamber. After incubation for 8 h at 37°C, nonmigratory cells on the upper membrane surface were removed with a cotton swab, and migratory cells migrating through the membrane pores and invading to the underside surface of the membrane were fixed with 4% paraformaldehyde and stained with Coomassie Brilliant Blue. For quantitative assessment, the number of stained, migrating cells was then counted under microscopy at five fields per filter in three independent experiments.

### Immunocytochemistry and F-actin staining

For immunocytochemical analysis, OECs or astrocytes on coverslips were washed with phosphate buffered saline (PBS) and fixed with 4% paraformaldehyde (PFA) in PBS for 20 min, followed by permeabilization with 0.3% Triton X-100 in 0.1 M PBS for 10 minutes. After blocking the non-specific binding with 10% normal goat serum in 0.1 M PBS, cells were incubated with primary antibodies against nestin (Chemicon), GFAP (Sigma), P75 (Chemicon), TNFR1 (Santa cruz), p-ERK (Santa cruz) or S100 (Boster) at 4°C overnight. Cells were then washed and incubated with fluorescence-conjugated secondary antibodies (Jackson ImmunoResearch Laboratories, Inc.) for 60 minutes at RT. For visualization of F-actin, cells were incubated with 0.1 µg/ml TRITC-phalloidin (Sigma) for 40 min at RT.

### Western blot

Astrocytes were rinsed briefly with ice-cold PBS and lysed for 5 min in SDS gel sample buffer. The cell lysates were denatured by boiling for 10 minutes and then centrifuged for 10 min at 13,000 g at 4°C. Proteins were separated by sodium dodecyl sulfate (SDS)-polyacrylamide gel and then transferred onto nitrocellulose membranes. Membranes were then blocked with 10% non-fat milk in 1× TBST and incubated with primary antibodies against TNFR1 (Santa cruz), TNF-α (R & D), pERK (Santa cruz) or ERK (Santa cruz). To control for differences in protein loading, membranes were also incubated with anti-GAPDH antibody (Sigma). After incubating with horseradish peroxidase (HRP)-conjugated secondary antibodies (Sigma), immunoreactive bands were visualized by chemiluminescence reagents (ECL, Amersham).

### RT-PCR

For RT–PCR, total RNA was extracted from cultured OECs using TRIZOL Reagent (Invitrogen Corporation, Carlsbad, CA), followed by the treatment with DNase I (DNA-free, Ambion, Austin, TX). Synthesis of cDNA was carried out with the Superscript First-Strand Synthesis System for RT-PCR (Invitrogen Corporation, Carlsbad, CA). The following primers were used for RT-PCR: TNFR1 forward primer (5′-GAACACCGTGTGTAACTGCC-3′) and reverse primer (5′- ATTCCTTCACCCTCCACCTC-3′); TNFR2 forward primer (5′-GATGAGAAATCCC AGGATGCAGTAGG-3′) and reverse primer (5′- TGCTACAGACGTTCACGATGCAGG-3′); ß-actin forward primer (5′-AAGATTTGGCACCACACTTTCTAC-3′) and reverse primer (5′-CACGGTTGGCCTTAGGGTT-3′). Aliquots (2 µl out of 20 µl reaction) of each PCR reaction were taken at 35 cycles and electrophoresed on a 1.0% agarose gel. Gels were stained with ethidium bromide and photographed under ultraviolet light.

### Immunohistochemistry

For immunohistochemical analysis, animals were deeply anaesthetized with 2% pentobarbital sodium and perfused transcardially with 4% PFA in 0.1 M PBS, pH 7.4. The spinal cords were subsequently dissected from each animal and post-fixed in the perfusing solution overnight at 4°C. Then, the tissues were cryoprotected in 20% sucrose in PBS for 24–48 h at 4°C. Cryostat sections (10 µm) were cut and mounted onto gelatin-subbed slides and stored at −70°C. For immunostaining, the slides were permeabilized and blocked with 0.3% Triton X-100/10% normal goat serum in 0.1 M PBS for 15 min. Primary antibodies against GFAP (Sigma), nestin (Chemicon) or TNF-α (R & D) were then applied to the sections overnight at 4°C. The following day, sections were incubated with fluorescence-conjugated secondary antibodies (Jackson ImmunoResearch Laboratories, Inc.) and examined by Olympus fluorescence microscopy.

### In vivo migration assay

To investigate whether reactive astrocyte-derived TNF-α affected the migration of implanted OECs, an in vivo migration assay was performed as previously described [18]. Cultured GFP-expressing OECs were harvested with 0.25% trypsin/1% EDTA when they reached 80–90% confluence. Cell suspensions were prepared at a concentration of 100,000 OECs/µL in DMEM and maintained on ice for transplantation. Immediately after completing the SCI model of rats, 0.5 µl of OECs were stereotaxically microinjected into the spinal cord dorsal column midline, 1.5 mm rostral to the lesion site using a pulled glass micropipette with an inner diameter of 40 µm. There were three transplantation groups: In group 1, untreated OECs were injected into spinal cord and received normal saline (N.S., 10 µl/day) through a pipe embedded in subarachnoid space (N.S. group). In groups 2, OECs pretreated with anti-TNFR1 antibody (1∶100) for 30 min were injected into spinal cord and received the antibody against TNFR1 (10 µl/day) instead of N.S. (TNFR1 group). In group 3, OECs pretreated with an irrelevant goat IgG (1∶100) for 30 min were injected into spinal cord and received the IgG (10 µl/day) through the pipe (IgG group).

Ten days after treatment, animals were killed and a 1 cm length of the spinal cord centered at the injury site was separated. For quantification of the migration of implanted OECs, longitudinal sections (10 µm) were cut in the horizontal plane. Every seven section was collected and the grafted GFP-expressing OECs were identified using a fluorescence microscope (IX70, OLYMPUS). Cell location was measured as the straight-line distance from the center of the injection site to the farthest OEC in the rostral and caudal directions. The maximum measured distance that the cells were located from the center of the lesion site of each animal was averaged within each group.

### Statistical analyses

All data presented represent results from at least three independent experiments. Statistical analysis was performed using Student's *t*-test or the Kolmogorov-Smirnov test. Statistical significance was defined as *P*<0.05.

## Results

### Glial scar tissue and reactive astrocytes promote OECs migration

**Figure 1:**
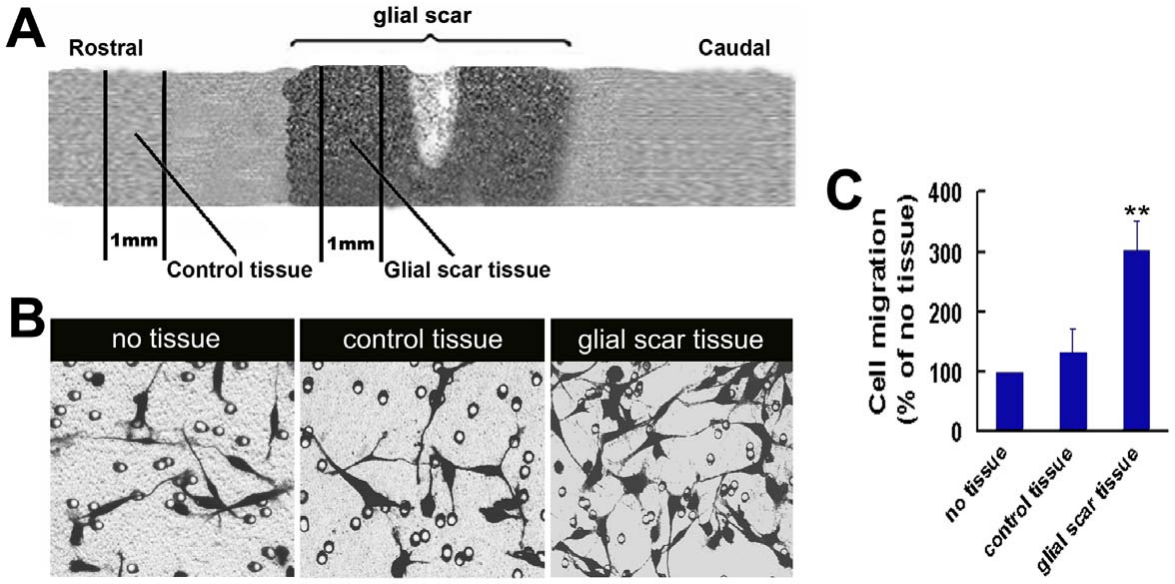
Analysis of the effect of glial scar tissue on OECs migration by a Boyden chamber migration assay. (A) Schematic representation of the control tissue and glial scar tissue prepared from injured spinal cord. Seven days after SCI, the spinal cord spanning lesion site was dissected and placed in Hank's balanced salt solution. Glial scar tissue was prepared by cutting an approximately 1-mm-thick cross-section of spinal cord in the region of glial scar. Control tissue was prepared by cutting an approximately 1-mm-thick cross-section of spinal cord distal to the region of glial scar. The mobility of OECs was analyzed in a Boyden chamber migration assay. OECs were seeded onto the upper chamber at a density of 4×10^5^ cells, with the spinal cord explants (control tissue or glial scar) plated into the lower chamber, and incubated 8 hr at 37°C. (B) Photomicrograph of OECs transmigrated through the filter in the absence or presence of glial scar tissue. (C) Quantitative assessment of migrating cells under different conditions. **P<0.01 versus control tissue group.

**Figure 2:**
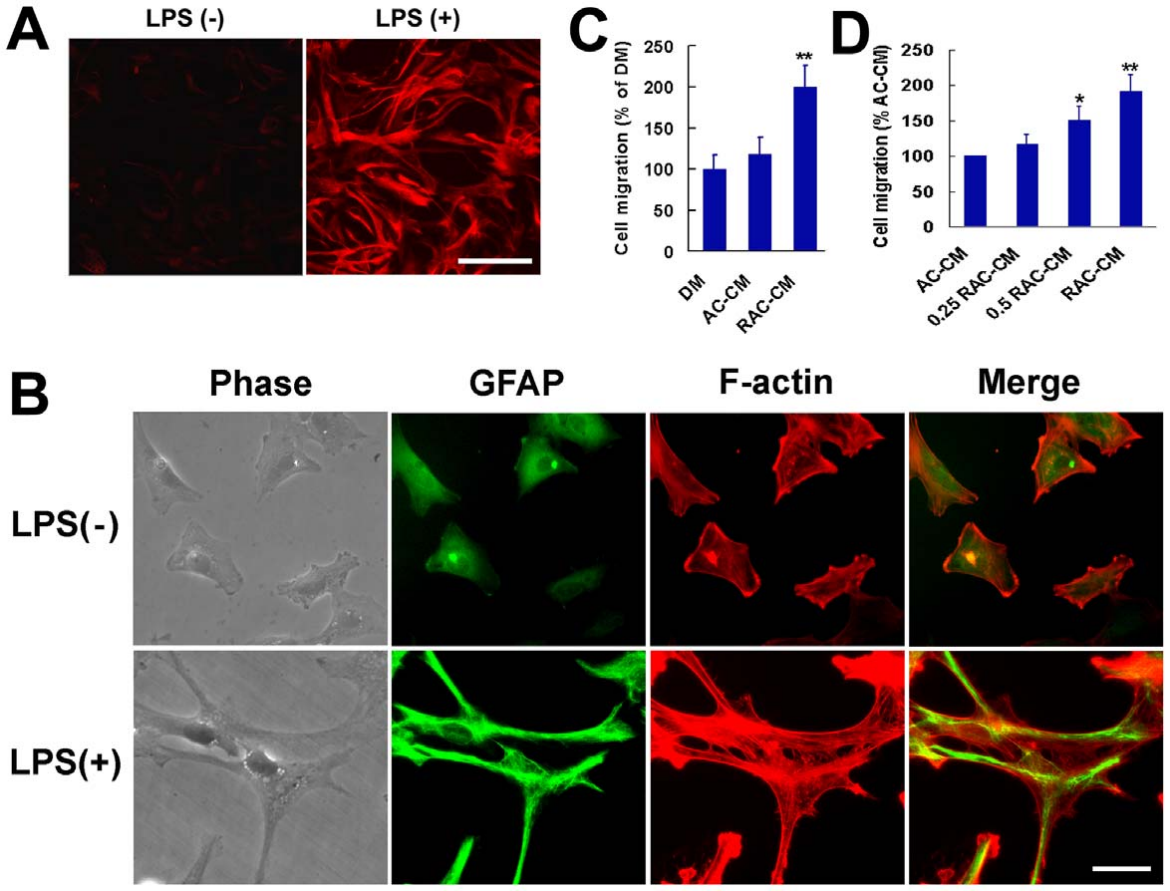
Reactive astrocytes-conditioned medium promotes OECs migration. (A, B) lipopolysaccharide (LPS) stimulates astrocytes to change into reactive astrocytes. After incubation with LPS (10 µg/mL) for 24 hr, astrocytes are activated to express nestin (A) and undergo an extensive hypertrophy of their cell bodies and cytoplasmic processes and a massive up-regulation of GFAP (B). Scale bars: 50 µm (A); 25 µm (B). (C) Reactive astrocyte-conditioned medium (RAC-CM) promotes OECs migration. After stimulation with defined medium (DM), astrocyte-conditioned medium (AC-CM) or RAC-CM for 8 hr, the migrating cells in the Boyden chambers are stained with Coomassie Brilliant Blue and counted. **P<0.01 versus DM. (D) Enhancement of migration of OECs by RAC-CM is dose-dependent. *P<0.05 versus AC-CM, **P<0.01 versus AC-CM.

To determine effects of glial scar on OECs migration, we measured the mobility of OECs by using a cell culture model of migration of OECs with co-cultured glial scar tissue in a Boyden chamber. The motility of OECs was determined by counting cells migrated through the filter which were stained with Commassie Blue. As shown in Fig. 1, co-culture with glial scar tissue but not control tissue promotes the migration of OECs. As the glial scar is composed primarily of reactive astrocytes induced by injury [2], we next examined whether the migration-promoting effect of glial scar tissue was mediated by reactive astrocytes. As shown in Fig. 2A and B, treatment of astrocytes with LPS resulted in stellation, extension of processes, re-expression of nestin, thickening of F-actin and more prominent expression of GFAP, typical of activated astrocytes. The reactive astrocyte-conditioned medium but not astrocyte-conditioned medium increased the motility of OECs in a concentration-dependent manner (Fig. 2C and D).

### Reactive astrocyte-derived TNF-α is responsible for the promoting effect on OECs migration

**Figure 3:**
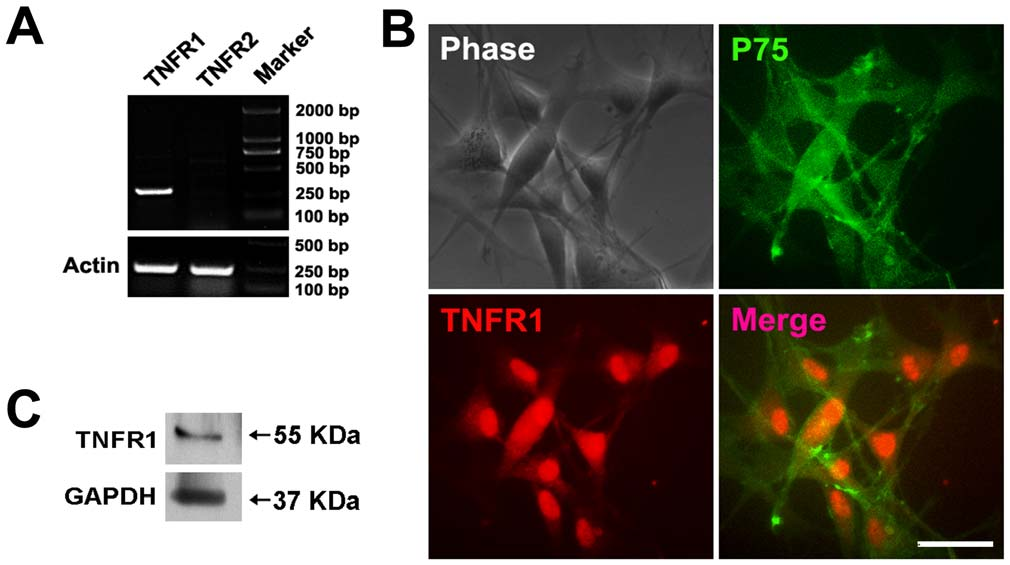
TNFR1 is expressed on OECs. (A) The mRNA of TNFR1 (301 bp) but not TNFR2 (264 bp) was identified in OECs by RT-PCR. (B) Cultured OECs were double-stained with antibodies against P75 and TNFR1 after treatment with Triton X-100. Scale bar = 50 µm. (C) Western blotting analysis for the expression of TNFR1 cell lysates of cultured OECs. GAPDH blotting severed as the loading control.

**Figure 4:**
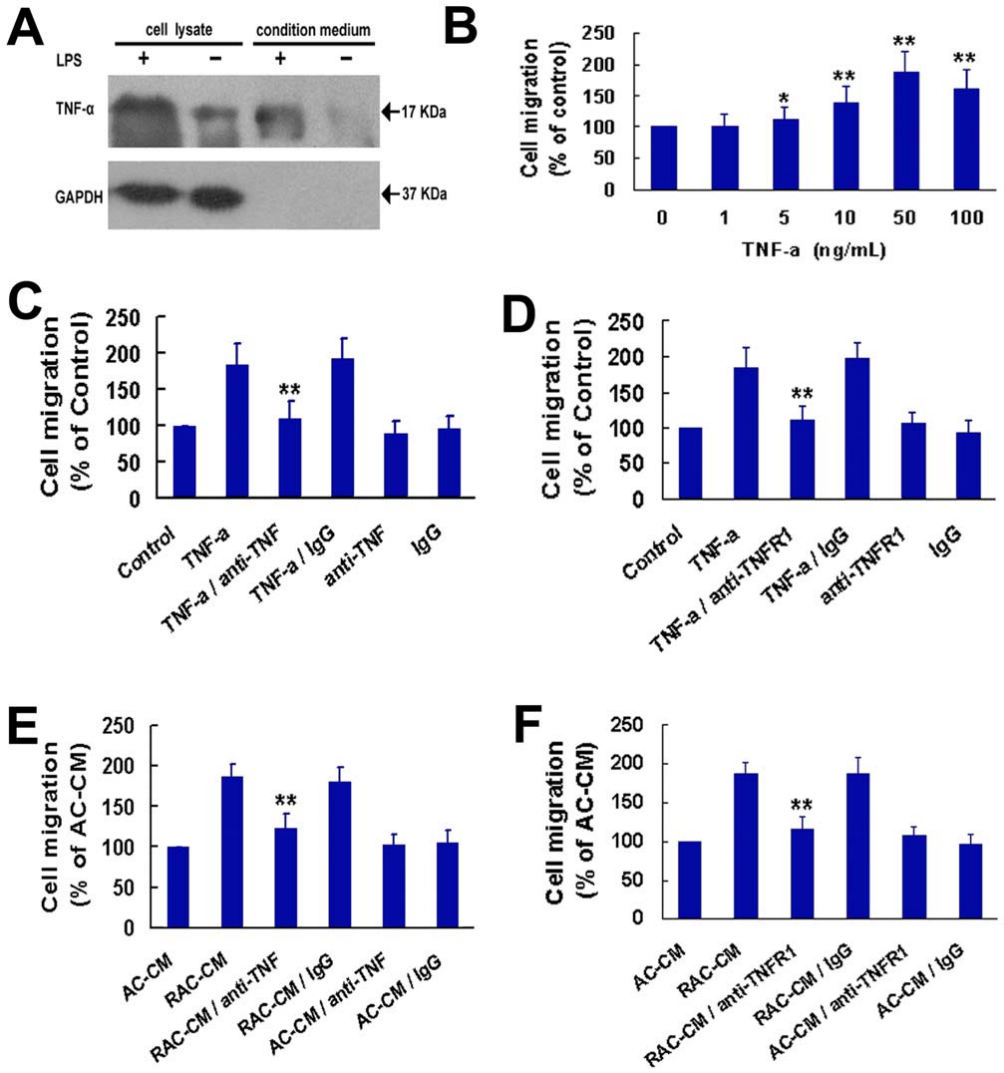
Reactive astrocytes secrete TNF-α to promote OECs migration by interacting with TNFR1. (A) Reactive astrocytes produce TNF-α. The cell lysate and condition medium of OECs treated (+) or untreated (−) with LPS (10 µg/mL) at 37°C for 24 hr were subjected to immunoblotting with antibody against TNF-α (upper panel) and anti-GAPDH (lower panel). (B) Dose dependency of TNF-α effect on OECs migration. *P<0.05 versus control, **P<0.01 versus control. (C, D) The effect of TNF-α on the migration of OECs is mediated by TNFR1. Medium or OECs were pretreated with TNF-α antibody (5 µg/mL), TNFR1 antibody (2 µg/mL) or goat IgG for 2 hr and applied to Boyden chamber migration assays, stimulated with TNF-α (50 ng/mL). Cells without pretreatment but with TNF-α stimulation were defined as TNF-α group. Cells with neither pretreatment nor TNF-α stimulation were defined as control. **P<0.01 versus TNF-α group. (E, F) TNF-α in RAC-CM is involved in mediating the migration-promoting activity of OECs. RAC-CM or OECs were pretreated with TNF-α antibody (5 µg/mL), TNFR1 antibody (2 µg/mL) or goat IgG for 2 hr, then stimulated with RAC-CM. Cells without pretreatment but with RAC-CM stimulation were defined as RAC-CM group. Cells without pretreatment but with AC-CM stimulation were defined as AC-CM group. **P<0.01 versus RAC-CM group.

When astrocytes were activated, they secreted a mass of inflammatory factors, such as TNF-α, IL-1β, IL-6, γ-IFN et al [25], [26], [27], [28], [29]. Since TNF-α was shown to influence the migration of various kinds of cells [30], [31], [32], [33], we next tested whether TNF-α released by reactive astrocytes was involved in mediating the migration-promoting activity of OECs. We first determined whether the receptors of TNF-α were expressed in OECs. Fig. 3A showed that expression of TNFR1 but not TNFR2 mRNA transcripts was detected in cultured OECs by RT-PCR analysis. The expression of TNFR1 in OECs was further confirmed by immunostaining and western blotting analysis (Fig. 3B, C). In addition, TNF-α was shown to exist in the condition medium of reactive astrocytes induced by LPS (Fig. 4A). Boyden chamber migration assay indicated that OECs migration was significantly promoted by recombinant TNF-α protein in a dose-dependent manner (Fig. 4B). When TNF-α antibody was added into the culture medium, recombinant TNF-α-induced enhancement of OECs migration was greatly attenuated (Fig. 4C). Antibody blocking against TNFR1 also abolished the migration promoting activity of recombinant TNF-α (Fig. 4D). Similarly, the ability of reactive astrocyte-conditioned medium to enhance the motility of OECs was significantly depressed by pretreatment of OECs with anti-TNFR1 or blocking TNF-α in medium with antibody (Fig. 4E, F). The motility of OECs was not influenced by treatment with blocking antibodies (anti-TNF-α or anti-TNFR1) alone or an irrelevant IgG, compared with the control (Fig. 4C–F). Together, these results strongly suggested that the enhancement of OECs migration was mediated by reactive astrocyte-derived TNF-α.

### TNF-α induced migration of OECs is MAPK-dependent

**Figure 5:**
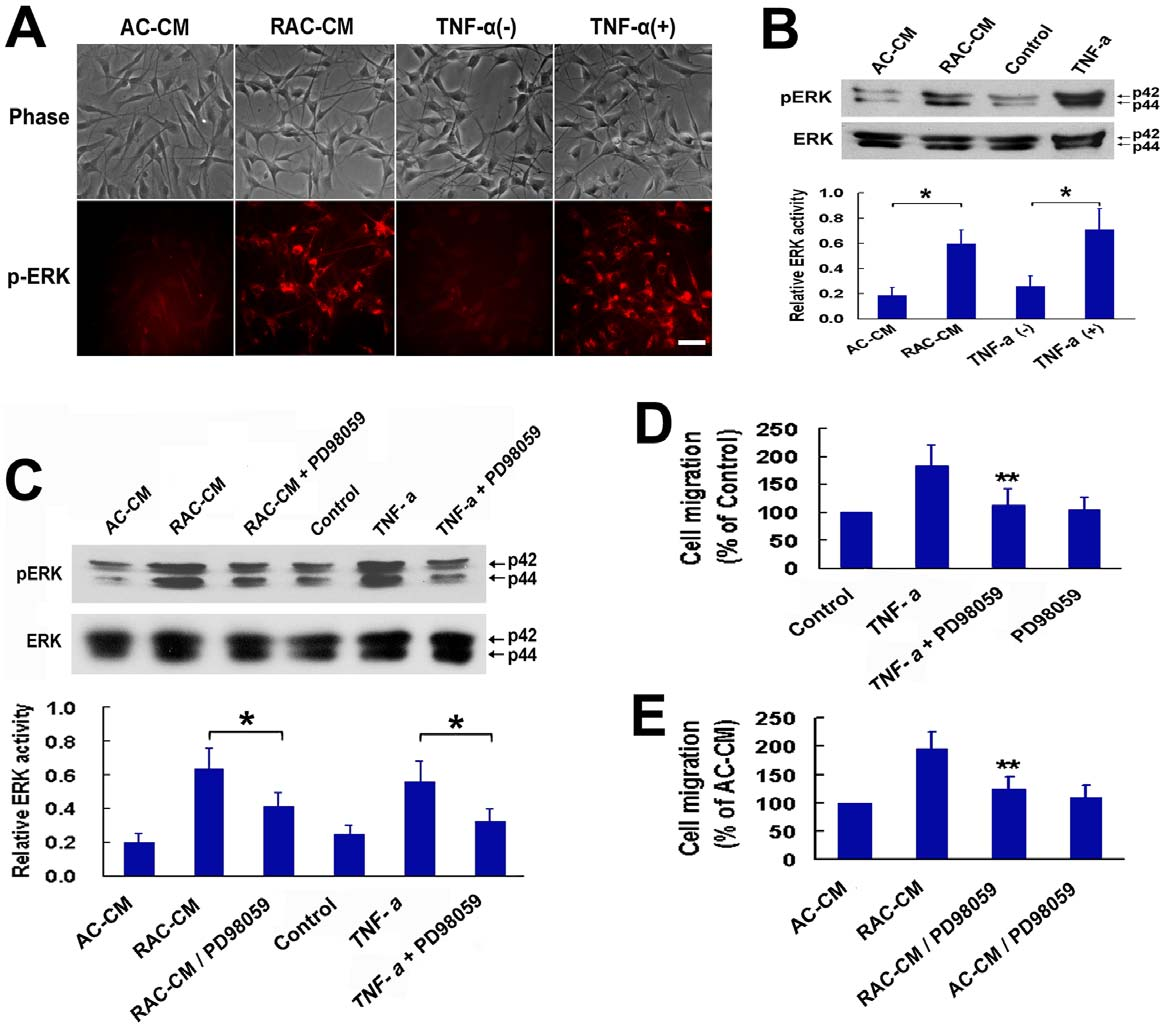
ERK activation is critical for mediating the effect of TNF-α on OECs migration. (A, B) ERK activation in OECs was assessed with immunocytochemistry (A) and western blot (B) after incubation with RAC-CM or recombinant TNF-α. Densitometric analysis is shown in the bottom panel of (B) with the amount of p-ERK normalized to the amount of ERK. *P<0.05. Scale bar = 50 µm. (C) PD98059 treatment significantly inhibited the activation of ERK in OECs stimulated with RAC-CM or recombinant TNF-α. OECs were incubated with ERK-inhibitor PD98059 (30 µM) for 30 minutes before RAC-CM or TNF-a (50 ng/mL) was added. Densitometric analysis is shown in the bottom panel. *P<0.05. (D, E) In Boyden chamber migration assay, the migration induced by RAC-CM or recombinant TNF-α was evidently attenuated when OECs were pretreated with PD98059. **P<0.01 versus TNF-α or RAC-CM.

Activation of the mitogen-activated protein kinase (MAPK) is known to be an important step in the migration of vascular smooth muscle cells [34], [35]. In addition, TNF-α has been shown to induce MAPK activation in several other cell types including fibroblasts [36], [37]. We therefore examined whether the MAPK pathway was involved in TNF-α–induced OECs migration. As shown in Fig. 5A and B, stimulation of OECs with reactive astrocyte-conditioned medium or recombinant TNF-α protein resulted in prominent activation of extracellular signal-regulated kinase 1/2 (ERK1/2). OECs grown in astrocyte-conditioned medium or serum-free medium demonstrated only weak ERK1/2 phosphorylation (Fig. 5A, B). Treatment of OECs with reactive astrocyte-conditioned medium or recombinant TNF-α induced a significant increase in the amount of phosphorylated ERK1/2, which was greatly attenuated by pretreatment of cells with PD98059, a selective MAPK inhibitor (Fig. 5B, C). In the migration assay, the promoting-effect of reactive astrocyte-conditioned medium on OECs migration was significantly blocked after pretreating these cells with PD98059 (Fig. 5D). Similarly, PD98059 also inhibited the response of OECs toward TNF-α (Fig. 5E). In addition, PD98059 alone did not change the migration activity of OECs compared with the controls (Fig. 5D, E). Together, these results suggested ERK signaling pathway was involved in mediating the effect of TNF-α on OECs migration.

### TNF-α secreted by reactive astrocytes in glial scar attracts OECs migration

**Figure 6:**
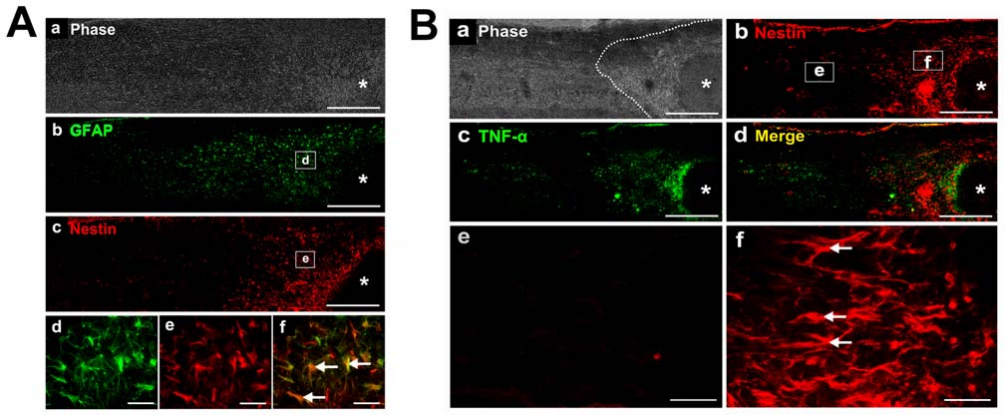
Astrocytes are activated to secrete TNF-α in hemisection SCI model. (A)Ten days after SCI, immunohistochemical analysis was performed for the reactive astrocytes with anti-GFAP and anti-nestin. (d, e) Higher magnification of the outlined areas in b and c showed reactive astrocyte with an extensive hypertrophy of their cell bodies and cytoplasmic processes. (f) A merged picture of d and e. Scale bars: 500 µm (a–c); 50 µm (d–f). (B) TNF-α produced by reactive astrocytes was examined by double-stained with antibodies against nestin and TNF-α 10 days after SCI (a–d). (e, f) Higher magnification of the outlined areas in b. Scale bars: 500 µm (a–d); 50 µm (e, f). Asterisk indicates the lesion site. Dashed line in B indicates approximate border of the glial scar. Arrows indicate representative reactive astrocytes.

To investigate the effect of reactive astrocytes in glial scar on OECs migration, we first detected the expression of TNF-α by reactive astrocytes in glial scar using a spinal cord hemisection model. Ten days after SCI, astrocytes surrounding the lesion epicenter underwent a typical change of hypertrophy, process extension and increased expression of intermediate filaments such as GFAP and nestin, characteristic of reactive astrocytes (Fig. 6A). By contrast, weak immunostaining for GFAP and nestin was observed in distant areas, without obvious changes in cellular morphology (Fig. 6A). Although TNF-α, a secretory protein, was not colocalized well with GFAP, Fig. 6B showed that spinal cord hemisection resulted in the expression of TNF-α and formed a linear concentration gradient with the highest fluorescent density in the region adjacent to the injury site (Fig. 6B). These data implied that TNF-α was released from activated astrocytes induced by CNS damage and the linear concentration gradient of TNF-α might be a chemoattractive cue for OECs migration toward the lesion site.

**Figure 7:**
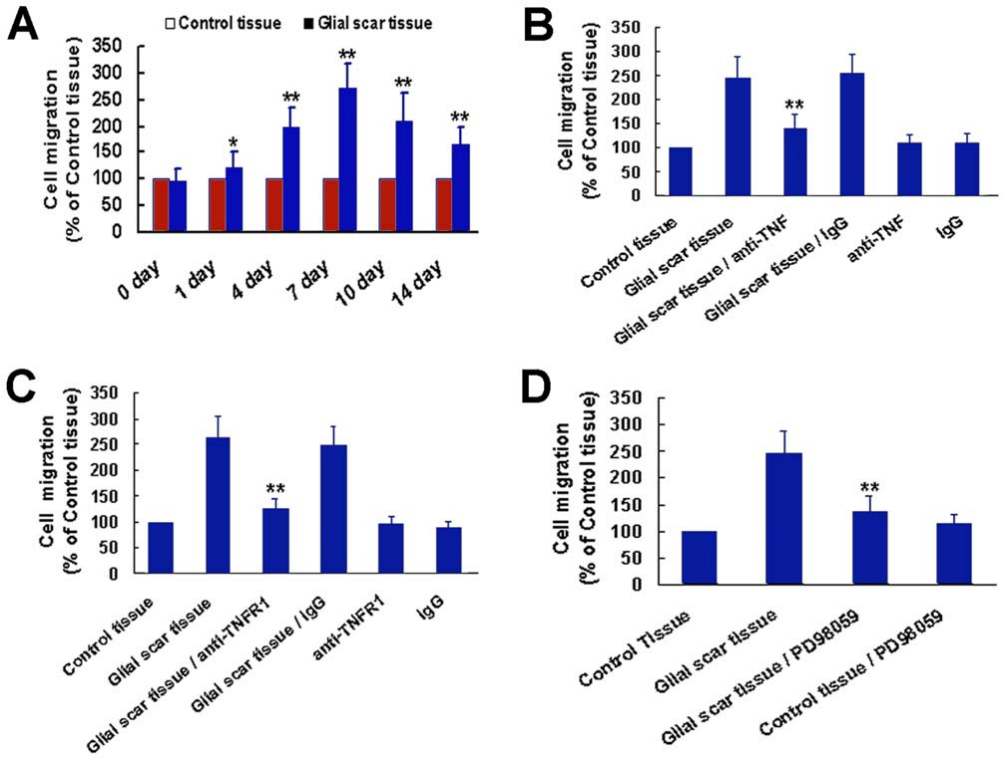
The effect of glial scar on OECs migration is mediated by secreted TNF-α. (A) Glial scar promotes OECs migration in a manner of time-dependence. *P<0.05 or **P<0.01 versus control tissue. (B, C) The effect of glial scar on OECs migration is mediated by TNF-α and TNFR1. OECs were pretreated with TNF-α antibody (5 µg/mL), TNFR1 antibody (2 µg/mL) or goat IgG for 2 hours, and then incubated with glial scar tissue in a Boyden chamber. Cells without pretreatment but with glial scar tissue stimulation were defined as glial scar tissue group. Cells without pretreatment but with control tissue stimulation were defined as control tissue group. **P<0.01 versus glial scar tissue. (D) ERK activation is involved in glial scar-induced OECs migration. PD98059 treatment significantly attenuated the effect of glial scar tissue on OECs migration. **P<0.01 versus glial scar tissue group.

Using the co-culture model of cells migration described above, we next examined the effect of glial scar prepared from injured spinal cord on OECs migration 0, 1, 4, 7, 10 and 14 days after SCI. As shown in Fig. 7A, the OECs migration-promoting effect of glial scar tissue was time-dependent. Promoting the mobility of OECs by glial scar tissue was observed as early as 1 day after SCI and reached a maximum at 7 days after SCI, but it subsequently decreased. When TNF-α secreted by reactive astrocytes in glial scar was neutralized by anti- TNF-α antibody or TNR1 expressed on OECs was blocked with anti-TNFR1, OECs migration induced by glial scar tissue was greatly attenuated (Fig. 7B, C). Pretreatment of OECs with ERK inhibitor PD98059 also significantly decreased the migrated cells induced by glial scar tissue (Fig. 7D). These results suggested that the effect of glial scar tissue on the motility of OECs was mediated by secreted TNF-α.

**Figure 8:**
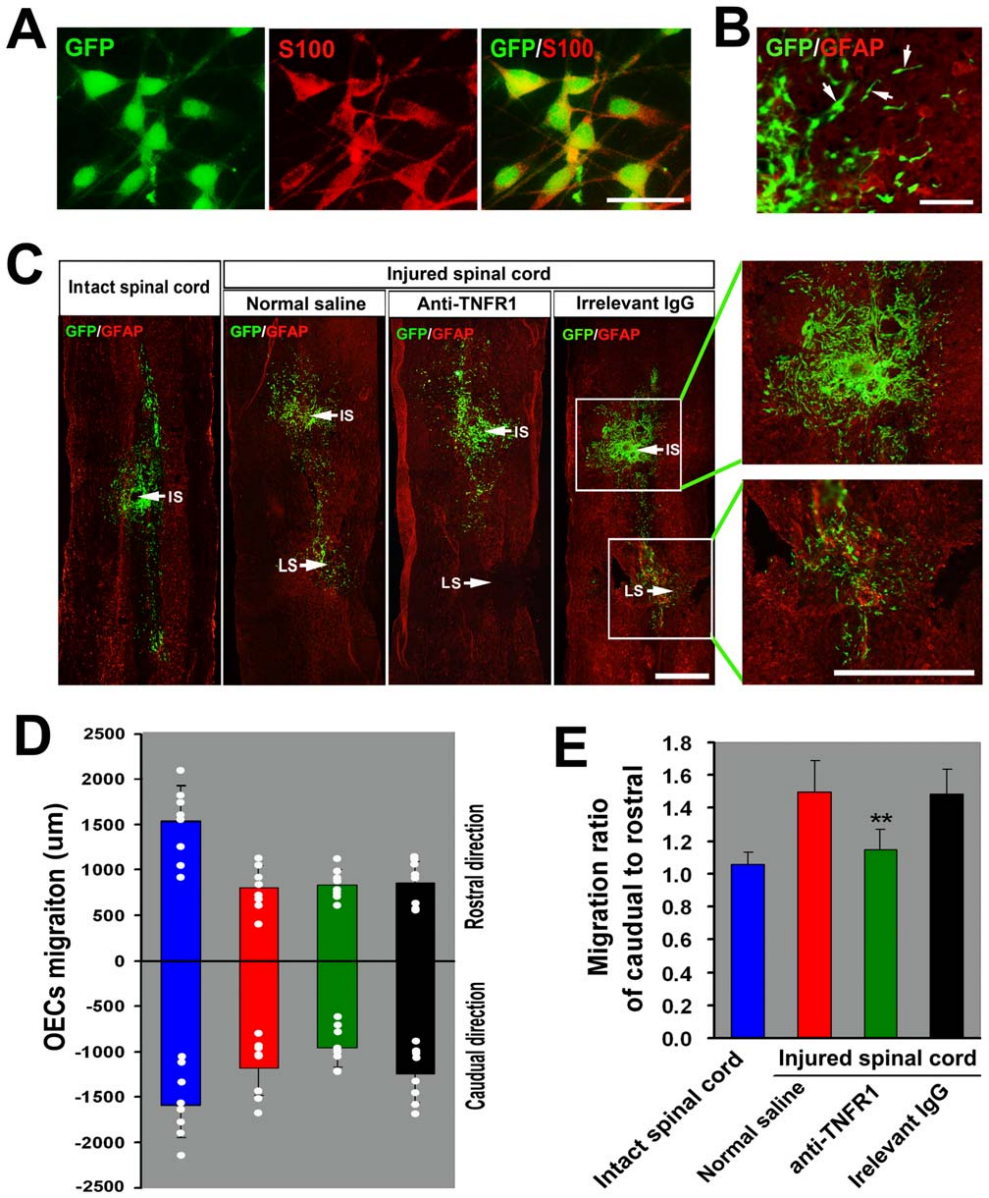
TNF-α attracts implanted OECs migration toward lesion site *in vivo*. (A) S100 immunolabeling of GFP-expressing OECs *in vitro*. The majority of OECs cultured from GFP-transgenic rats express S100. (B) Higher power showing OECs at the edge of the injection site. Elongated profiles (arrows), suggestive of cell migration, as well as round and multipolar GFP labelled cells can be seen. (C) Horizontal sections from intact and contralateral hemisectioned spinal cords with GFP-expressing OECs injections 1.5 mm rostral to the lesion epicenter at the time of injury. The animals with SCI were treated with normal saline (N.S.), anti-TNFR1 antibody or irrelevant goat IgG every day through a pipe embedded in the subarachnoid space. Arrows indicate the injection site (IS) and lesion site (LS). (D) Quantitative assessment of cell migration at 10 days after transplantation into the spinal cord. Mean and standard deviation of the migration distances in rostral and caudal directions are presented for each group. The white dot indicates the migration distance for each injection. Blue bars, OECs into intact spinal cord; red bars, OECs into injured spinal cord and treated with normal saline; green bars, OECs into injured spinal cord and treated with anti-TNFR1; black bars, OECs into injured spinal cord and treated with irrelevant IgG. (E) Histogram showing the average migration ratio of the caudal to rostral direction. **P<0.01 versus normal saline or irrelevant IgG group. Scale bars: 50 µm (A), 100 µm (B), 500 µm (C).

We further determined whether TNF-α could attract implanted OECs migration toward the lesion site *in vivo*. Immunocytochemistry revealed that 96.8±0.5% of GFP-expressing OECs were labeled for the OECs marker S100 before grafting (Fig. 8A). After transplantation 1.5 mm rostral to the spinal cord injury site, the GFP labelled cells were predominantly elongate and bipolar, suggestive of cell migration, near the edge of the area of injected cells (Fig. 8B). As shown in Fig. 8C, grafted OECs migrated away from the injection site. In uninjured spinal cord, there was no significant difference in the migration distance between rostral and caudual direction (Fig. 8C–E). Although OECs migration distance was reduced in hemisected spinal cord, compared with the non-injured spinal cord, there was a tendency of cells to move farther in the caudal direction (toward the lesion site) than in the rostral direction (Fig. 8C–E). However, treatment of OECs with anti-TNFR1, but not normal saline or an irrelevant IgG, inhibited the preferential caudal migration (Fig. 8C–E). Taken together, these data suggested that TNF-α released from activated astrocytes induced by SCI attracted OECs migrating toward lesion site *in vivo*.

## Discussion

Owing to the lack of axonal regeneration and reconnection with correct synaptic targets, the repair of CNS damage, especially SCI, continues to be a major challenge. Because of the existence of glial scar in damaged CNS, severed axons are difficult to regenerate pass the lesion site. It has been reported that long-distance axonal regeneration beyond the lesion centre in the transected adult spinal cord is induced by transplantation of OECs [20], [38], [39]. OECs transplantation can inhibit hypertrophic response of host astrocyte, reduce CSPG expression in reactive astrocytes, and negatively regulate glial scar formation [21], [40], [41], [42]. Several studies report that OECs induce an increase in the formation of new blood vessels after SCI [20], [43], [44], [45], which may function as a scaffold for migrating glia as well as for regrowing axons [44]. Data from Li et al. [46] demonstrate that OECs actively phagocytose degenerating axons. Recent studies indicate that OECs form a channel- or tube-like structure within the lesion to provide an oriented matrix for axons to grow on or through [15], [17]. It is noteworthy that the migratory properties of OECs are considered to account in part for their “repair” qualities, as OECs are found to migrate and associate with extending axons and are proposed to provide permissive conditions for axon bridging beyond the injury site [20], [38], [47], [39]. Importantly, grafted OECs are shown to migrate preferentially toward the lesion site in the damaged CNS [18]. In the present study, data from *in vitro* assays showed that both LPS-induced reactive astrocytes and glial scar tissue promote the migration of cultured OECs. Furthermore, in the spinal cord contralateral hemisection model, OECs injected rostral to lesion site were shown eventually to migrate from the site of grafting centripetally toward the glial scar and into lesion epicenter.

It has been proposed that OECs might respond to signals from the damage epicenter, which account for their migrating preferentially toward the injury site [38], [48]. Astrocytes have the capacity to secrete or respond to a variety of cytokines including IL-1, IL-6, IL-3, and TNF-α. Evidences show that TNF-α, an important inflammatory factor, regulates the migration of various kinds of cells. TNF-α and TNFR1 are required for migration of follicular dendritic cell precursors into splenic follicles [30]. Studies of Dekaris et al. [31] demonstrated that TNF-α regulates corneal Langerhans cell migration. Additionally, TNF-α has also been reported to induce the motility of intestinal epithelial cell and mammary epithelial cells [32], [33]. As a unique glial type in olfactory system, OECs has been shown to share some characteristics of inflammatory cell [49], [50], [51], [52], [53]. For example, data from recent microarray analysis of the transcriptome of OECs document that the cell, compared to astrocytes and Schwann cells, express higher levels of a number of innate immune factors, which suggest that OECs may play a role in modulating neuroinflammation [54]. OECs are also demonstrated to possess the cellular machinery that permits them to respond to certain bacterial ligands, and to mount a response through the activation of nuclear factor κB (NFκB), an inflammatory transcription factor, and cytokine production [52]. In this study, TNF-α was found existing in the culture medium of activated astroyctes induced by LPS. In the injured spinal cord, expression of TNF-α by reactive astroyctes in glial scar was up-regulated and formed a linear concentration gradient. Moreover, Boyden chamber migration assay indicated that TNF-α was a chemoattractant for OECs migration, and TNF-α blocking with antibody greatly decreased the OECs migration activity. In addition, TNFR1 (type 1 TNF-α receptor) but not TNFR2 (type 2 TNF-α receptor) was shown to express in OECs, and we further confirmed that the chemoattractive activity on the migration of OECs was TNFR1-dependent by using anti-TNFR blockage. To our knowledge, this is the first evidence that functional TNFR1 is expressed on OECs. All these results supported the hypothesis that in the injured spinal cord, reactive astrocytes in glial scar release TNF-α to attract grafted OECs migrating from injection site into lesion centre.

TNF-α has been shown to activate a number of signaling pathways in various cell types [55], [56], [57] and these signaling mechanisms are highly dependent on the cell type. It is reported that the MAPK pathway is activated by TNF-α in fibroblasts [37] and inflammatory cells [36]. TNF-α is shown to increase granulosa cells proliferation by activating stress-activated protein kinase/c-Jun-NH2-teminal kinase signaling [58]. In addition, evidences from Sippy et al. indicate that induction of intercellular adhesion molecule-1 by TNF-α is dependent on protein kinase C in human retinal pigment epithelial cells [55]. In present study, we found that the amount of active ERK1/2 was significantly increased in OECs treated with reactive astrocyte-conditioned medium or recombinant TNF-α, which suggested that TNF-α binding to TNFR1 led to activation of ERK1/2 in OECs. PD98059 selectively inhibits the dual-specificity kinase MEK, which phosphorylates and activates MAPK. Pretreating OECs with this pharmacological inhibitor blocked the activation of ERK1/2 and greatly attenuated the migration of these cells induced by reactive astrocyte-conditioned medium, glial scar tissue, or recombinant TNF-α. All these findings suggested that activation of ERK1/2 MAPK was a crucial event in TNF-α–directed migration of OECs.

There were controversial reports regarding migration of transplanted OECs in the injured spinal cord. Some studies show that OECs readily coexist with reactive astrocytes and are able to pass through this barrier when injected at the time of injury and after a delay, following scar formation [15], [17], [21], [40], [59], [60]. Reactive astrocytes, present in all type of injured cases do not appear to limit the movement of OECs even if the glial scar had already formed prior to OEC implantation. However, data from other study demonstrate that the motility of grafted OECs in damaged CNS is limited, with no OECs found migrating into glial scar [19]. It is likely that the difference in the injection site and the time for transplantation may account for the conflicting reports about OECs migration in damaged spinal cord. As shown in the present study, after SCI, a linear concentration gradient of TNF-α is formed in the glial scar. If OECs are transplanted in a site far away from lesion centre, the concentration of TNF-α may be too low to attract OECs migrating into glial scar. Therefore,an appropriate injection site is crucial for the grafted OECs to response to signals or factors from glial scar. Previous studies demonstrated that the functional improvement was achieved by delayed transplantation of OECs [61], [62], [63]. Interestingly, our data showed that the chemoattractive activity of glial scar tissue on OECs migration is time-dependent, with a maximal activity at 7 days after SCI. This finding may be beneficial for guiding the selection of time point for OECs transplantation after SCI.

In summary, our study provide the first evidence that TNF-α released by reactive astrocytes attracts OECs migration. This work suggests a molecular mechanism underlying OECs migration into glial scar and indicates a novel function of reactive astrocytes in OECs-based treatment for CNS injury.
